# The Association of eNOS Gene Polymorphism with Avascular Necrosis of Femoral Head

**DOI:** 10.1371/journal.pone.0087583

**Published:** 2014-02-03

**Authors:** Liwen Zheng, Wanchun Wang, Jiangdon Ni, Zhihong Li, Tao Xiao

**Affiliations:** Department of Orthopedics, 2^nd^ Xiangya Hospital, Central South University, Changsha, Hunan, PR China; The Scripps Research Institute, United States of America

## Abstract

**Objectives:**

Necrosis of femoral head is a severe pathological state with multiple etiologies. This study investigated the association of the 27-bp repeat polymorphism in intron 4 and G894T polymorphism in exon 7 of the endothelial nitric oxide synthase (eNOS) gene with the pathogenesis of avascular necrosis of femoral head (ANFH).

**Methods:**

A total of 125 non-traumatic ANFH patients and 126 healthy controls were recruited for this study. The 27-bp repeat polymorphisms in intron 4 were analyzed by polymerase chain reaction (PCR) and sequencing. The G894T polymorphisms in exon 7 were analyzed by PCR– restriction fragment length polymorphism (PCR-RFLP) analysis.

**Results:**

All alleles were observed in non-traumatic ANFH patients and control subjects. Both ANFH patients and idiopathic subgroup of ANFH patients showed higher frequency of the 4a/b genotype than controls (p  = 0.001 and p  = 0.020, respectively). Significantly higher frequency of G/T genotype was observed in ANFH patients and idiopathic subgroup of ANFH patients compared to controls (p  = 0.009 and p  = 0.035, respectively).

**Conclusion:**

eNOS gene polymorphisms may be a risk factor for ANFH. The 27-bp repeat polymorphism in intron 4, G894T polymorphism in exon 7, and subsequently reduced eNOS activity may be involved in the etiology of idiopathic ANFH.

## Introduction

Avascular necrosis of femoral head (ANFH) is a pathological state with multiple etiologies that reduce vascular supply to the subchondral bone of the femoral head, resulting in osteocyte death and progressive collapse of the articular surface followed by degenerative arthritis of the hip joint [1]. The accurate prevalence of ANFH is unknown because many early stage cases are not diagnosed, while many late stage cases can be misdiagnosed as osteoarthrosis. Even so, about 10,000 to 20,000 new cases are diagnosed in the United States each year [2]. ANFH is a common disease in China [3], but accurate epidemiological data are unavailable. This disease frequently occurs in adult patients between the ages of 20 and 50 years old [4]. The risk factors for 75%–90% of cases include chronic steroid use, alcoholism, smoking, hip trauma, and prior hip surgery [1]. Recent studies demonstrated that eNOS may be associated with a risk of developing idiopathic osteonecrosis [5].

Nitric oxide (NO) is a biomolecule that is involved in a variety of physiologic processes, including angiogenesis, thrombosis, and bone turnover, which have been proven to be related to the pathogenesis of osteonecrosis [6]. NO is produced by NO-synthases (NOS), which catalyze the conversion of L-arginine to L-citrulline and NO. There are three isoforms of NOS: neuronal (nNOS), induced (iNOS) and endothelial (eNOS) [7]. It has been reported that eNOS is the predominant isoform of NOS expressed in normal adult bone. The reduction in the expression or activity of eNOS could result in lower production of NO and subsequent abnormalities in vascular reactivity, platelet recruitment, angiogenesis, bone volume, and bone formation rate [8]. It has been demonstrated that two polymorphic sites of the eNOS gene, the 27-bp repeat polymorphism in intron 4 and G894T polymorphism in exon 7, are associated with the altered function of this gene [9]–[11]. A study in Korean patients demonstrated that polymorphism in intron 4 of eNOS was significantly associated with idiopathic femur head osteonecrosis (FHON), but the distribution of G894T polymorphisms was not significantly different between patients and controls [4]. The T786C polymorphism is related to the G894T polymorphism and was also reported to be associated with eNOS gene function [12]. Glueck et al. study in Caucasian and African American patients demonstrated that the T786C eNOS polymorphism and resultant reduction of nitric oxide production are associated with the pathogenesis of idiopathic ANFH [5]. Although only a few studies investigated the association of eNOS polymorphism with the pathogenesis of ANFH, the effects of genetic differences between populations and confounders could not be excluded. Currently, no report has investigated the association between eNOS polymorphisms and pathogenesis of ANFH in Chinese patients.

In this study, we investigated the 27-bp repeat polymorphism in intron 4 and G894T polymorphism in exon 7 of the eNOS gene in 125 Chinese patients with non-traumatic ANFH and evaluated ANFH according to the etiologic factors.

## Materials and Methods

### Subjects

One hundred and twenty five patients with non-traumatic ANFH (57 males and 68 females with an average age of 56.4±9.2 years) were consecutively enrolled in this study at the Department of Orthopedics from January, 2011 to November, 2012. ANFH was diagnosed and staged according to the Ficat system [13]. The diagnosis of stage 2, 3, and 4 disease was established by evidence of osteonecrosis on plain radiographs. The diagnosis of stage 1 disease was established by magnetic resonance imaging scans because plain radiographs are not sensitive enough for stage 1. According to etiologic factors, patients were subgrouped into idiopathic (65 cases), steroid-induced (27 cases), and alcohol-induced osteonecrosis (33 cases). Patients with a history of taking prednisolone ≥1800 mg or equivalent for over 4 weeks were categorized under steroid-induced osteonecrosis [14], [15]. Patients with a history of ethanol consumption of at least 800 mg per week were categorized under alcohol-induced osteonecrosis [16]. Patients with ANFH that was caused by direct trauma and patients with ANFH concurrent with cardiovascular diseases, such as atherosclerosis, arterial thrombosis, HIV infection, smoking, diabetes mellitus, or renal dysfunction were excluded. One hundred and twenty six age and sex matched healthy controls (61 males and 65 females with an average age of 57.9±10.1 years) with no history of smoking were recruited from patients attending routine medical checkups. All participants were Han Chinese from Southwest China. The characteristics of the patients are summarized in Table 1. The study protocol was approved by the Ethics Committee of 2^nd^ Xiangya Hospital. Written informed consent was obtained from all participants or his/her guardians.

**Table 1:** Clinical etiological classification of ANFH patients.

		ANFH			
	Healthy Control(N = 126)	Total(N = 125)	Idiopathic(N = 65)	Steroid-induced(N = 27)	Alcohol-induced(N = 33)
Sex					
Male	61	57	33	6	18
Female	65	68	32	21	15
Age(year)	58.9±10.5	56.4±9.2	49.2±12.5	62.6±7.5	64.7±8.1
COD	n/a	79.1±46.5	42.5±20.8	101.5±76.4^a^	132.7±56.7^b^
Clinical Stage					
I	n/a	13	8	1	4
II	n/a	35	19	9	7
III	n/a	32	15	7	10
IV	n/a	45	23	10	12

DOD: duration of disease.^ a^p<0.0001,

b p<0.0001 *vs.* idiopathic group.

### DNA Isolation and Genotyping

**Figure 1:**
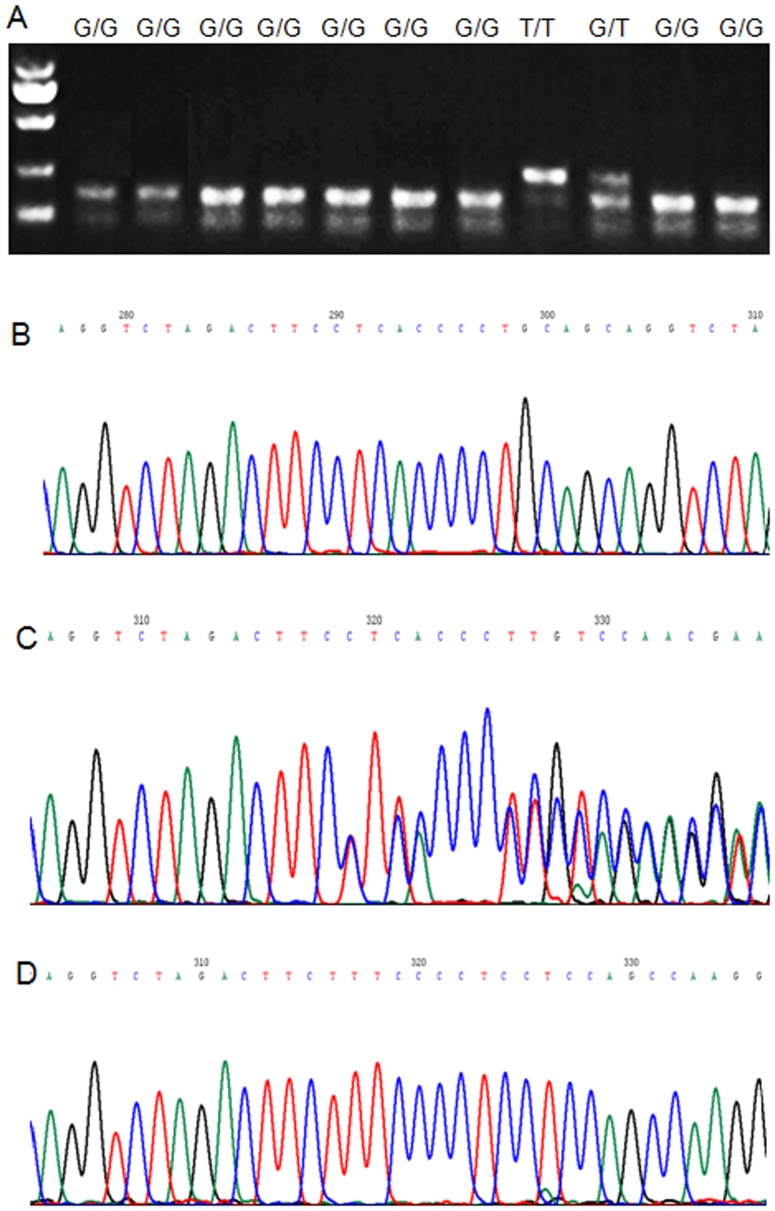
eNOS gene polymorphism analysis. A) PCR-RFLP analysis of exon 7 PCR product. B) Allele b/b of eNOS intron 4. C) Allele a/b of eNOS intron 4. D) Allele a/a of eNOS intron 4.

Genomic DNA was extracted from peripheral mononuclear cells using the DNeasy Tissue Kit (Qiagen, Valencia, CA). PCR amplification of 27-bp repeat polymorphism in intron 4 was performed using forward primer: 5 ′-AGGCCCTATGGTAGTGCCTT-3′ and reverse primer: 5 ′-TCTCTTAGTGCTGTGGTCAC-3′. The PCR products were separated on 1.5% agarose gels. PCR amplification of wild-type intron 4 containing five 27 bp repeats (the b allele) yields a 420 bp fragment, while amplification of mutant intron 4 containing four 27 bp repeats (the a allele) yields a 393 bp fragment. The PCR products were purified and sequenced. The presence of G894T variants in exon 7 was determined by polymerase chain reaction – restriction fragment length polymorphism (PCR-RFLP) analysis. The DNA containing G894T variants was amplified using forward primer: 5′-AAGGCAGGAGACAGTGGAGGT-3 and reverse primer: 5 ′-CCCAGTCAATCCCTTTGGTGCTCA-3′ and a 248 bp fragment was produced. Using the BanII restriction enzyme, the wild-type G894T will be cleaved into 163 bp and 85 bp fragments (GG). The mutated G894T will not be cleaved (TT). Only a 248 bp fragment and GT allele showed 165 bp, 85 bp and 248 bp fragments. DNA fragments were separated by electrophoresis on 1.5% agarose gels stained with ethidium bromide (Fig. 1).

### Statistical Analysis

Data were presented as mean ± standard deviation (X±SD) and analyzed using SPSS 18.0 software (SPSS Inc, Chicago, IL, USA). The Odds ratios were calculated and a logistic regression was used to obtain adjusted Ors and 95% CIs. Comparisons between variables were performed using Chi-squared test or paired samples *t*-test. A *p*<0.05 was considered statistically significant.

## Results

### Demographic Characteristics of Patients and Controls

The duration of disease in 125 non-traumatic ANFH patients ranged from 3 months to 21.5 years with an average of 107.2±67.3 months. The average disease duration in idiopathic ANFH patients was significantly shorter than that in alcohol-induced ANFH patients (p<0.001) and steroid-induced patients (p<0.001). No significant difference in age or sex was observed between patients and controls as well as in idiopathic and alcohol-induced ANFH. In contrast, significantly more female patients with steroid-induced ANFH than male patients were observed (Table 1).

### eNOS Polymorphisms are Associated with ANFH

All possible alleles were observed in non-traumatic ANFH patients and control subjects. Table 2 and 3 show the distribution of eNOS gene polymorphisms in both groups. The frequency of 4a allele was significantly higher in ANFH patients and idiopathic subgroup of ANFH patients than in the controls (9.2%, 11.5% *vs.* 2.4%, p<0.001, OR  = 4.105, 95%CI  = 1.504–10.433 and p<0.001, OR  = 4.524, 95%CI  = 1.733–11.507, respectively). Similarly, the frequency of 894T was significantly higher in ANFH patients and idiopathic subgroup of ANFH patients than in the controls (14.4%, 16.1% *vs.* 9.1%, p  = 0.037, OR  = 2.873, 95% CI  = 1.301–8.257 and p  = 0.042, OR  = 2.720, 95% CI  = 1.486–9.413, respectively) (Table 2). Meanwhile, total patient and idiopathic ANFH subgroup showed higher frequency of the 4a/b genotype than the control group (15.2%, 16.9% *vs.* 4.8%, p<0.001, OR  = 4.507, 95% CI  = 1.574–13.596 and p  = 0.020, OR  = 2.975, 95% CI  = 1.374–9.711, respectively). The frequency of G/T genotype in ANFH patients (24.0%), subgroup patients (23.1%, 23.6%, and 24.2% for idiopathic, steroid-induced, and alcohol-induced ANFH, respectively), and controls (13.5%) was calculated. Significant differences were only observed between total ANFH patients (p  = 0.009, OR  = 3.804, 95% CI  = 1.345–10.563) and idiopathic subgroup of ANFH patients (p  = 0.035, OR  = 2.205, 95% CI  = 1.123–8.605) and controls (Table 3).

**Table 2:** Allele Frequencies of Endothelial Nitric Oxide Synthase Gene Polymorphisms.

		ANFH			
Allele	Healthy Control N = 252(%)	Total N = 250(%)	Idiopathic N = 130(%)	Steroid-induced N = 54(%)	Alcohol-induced N = 66(%)
27 bp repeat polymorphism in intron 4					
4a	6(2.4%)	23(9.2%)^a^	15(11.5%)^b^	4(7.4%)^c^	4(6.1%)^d^
4b	246(97.6%)	227(90.8%)	115(88.5%)	50(92.6%)	62(93.3%)
G894T polymorphism in exon 7					
894T	23(9.1%)	36(14.4%)^e^	21(16.1%)^f^	7(13.0%)^g^	8(12.1%)^h^
894G	229(90.9%)	214(95.6%)	109(83.9%)	47(87.0%)	58(87.9%)

a p = 0.001, ^b^p<0.001, ^c^p = 0.059, ^d^p = 0.127, ^e^p = 0.037, ^f^p = 0.042, ^g^p = 0.390, ^h^p = 0.465 *vs.* health control.

**Table 3:** Genotype Frequencies of Endothelial Nitric Oxide Synthase Gene Polymorphisms.

		ANFH			
Allele	Healthy Control N = 126(%)	Total N = 125(%)	Idiopathic N = 65(%)	Steroid-induced N = 27(%)	Alcohol-induced N = 33(%)
27 bp repeat polymorphism in intron 4					
b/b	120(95.2%)	104(83.2%)	52(80.0%)	23(85.2%)	29(87.8%)
b/a	6(4.8%)	19(15.2%)^a^	11(16.9%)^c^	4(14.8%)	4(12.1%)
a/a	0(0)	2(1.6%)	2(3.1%)	0(0)	0(0)
G894T polymorphism in exon 7					
G/G	106(84.1%)	92(73.6%)	47(72.3%)	20(74.1%)	25(75.8%)
G/T	17(13.5%)	30(24.0%)^b^	15(23.1%)^d^	7(23.6%)	8(24.2%)
T/T	3(2.4%)	3(2.4%)	3(4.6%)	0(0)	1(4.8%)

a p<0.001, ^b^p = 0.0094, ^c^p = 0.0203, ^d^p = 0.0357 *vs.* healthy control.

## Discussion

Although non-traumatic ANFH has been widely recognized as a pathological state with multiple etiologies, the exact pathogenesis of osteonecrosis remains to be elucidated. Chronic steroid use and alcoholism were thought to be the main risk factors for osteonecrosis. Recent research has explored associations between genetic mutations, polymorphisms, and pathogenesis of ANFH. Single nucleotide polymorphisms in the multidrug resistance gene have been revealed to be associated with corticosteroid-induced osteonecrosis [17]. Genetic variation of alcohol-metabolizing enzyme genes has been associated with alcoholism-induced osteonecrosis [18]. Polymorphism in intron 4 and T786C polymorphism of eNOS were found to be significantly associated with idiopathic ANFH [4], [5]. In this study, we demonstrated that the frequency of 4a allele, b/a genotype in intron 4 and allele 894T, GT genotype in exon 7 were significantly higher in idiopathic subgroup of ANFH patients than in healthy controls.

It has been demonstrated that the presence of 27-bp repeat polymorphism in intron 4 and G894T polymorphism in exon 7 could result in a change in eNOS expression and enzymatic activity. For example, plasma NO levels in subjects with the 4a allele of eNOS were significantly lower than those without the 4a allele [11]. eNOS isoforms are processed differently within the cell depending on the presence of aspartate or glutamate at position 298 of the eNOS protein [8], [10]. Therefore, 4a and G894T gene polymorphisms can lead to low expression and activity of eNOS. Koo et al study in Korean patients demonstrated that the frequency of 4a allele and 4a/b genotype was significantly higher in femoral head osteonecrosis (FHON) patients and idiopathic subgroup of FHON patients compared to healthy controls. In contrast, the distribution of G894T polymorphisms was not significantly associated with FHON [4]. Consistent with the study in Korean patients, we observed that the frequency of 4a allele and 4a/b genotype was significantly higher in ANFH patients, especially in idiopathic osteonecrosis patients than in controls. In contrast, our study revealed that G894T polymorphism was significantly higher in ANFH patients as well as idiopathic osteonecrosis patients compared to controls. By considering that the sample size in our study (n = 125) matches the sample size (n = 103) in Koo et al study, the different findings between our studies may reflect genetic differences between populations. In addition, the samples' homogeneity may also contribute to the different findings. In Koo et al study, FHON patients with a history of cardiovascular diseases, HIV infection, smoking, diabetes mellitus, and renal disease were not excluded from their study. These cofounders have been widely demonstrated to be risk factors of FHON. In contrast, these cofounders were excluded from our study. Therefore, our study may be more accurate in reflecting the involvement of eNOS polymorphism in the pathogenesis of ANFH. Our study suggests that 4a/b genotype in intron 4 and G894T polymorphism in exon 7 may be a risk factor for ANFH, and NO produced by constitutively expressed eNOS may play a protective role in the pathogenesis of ANFH.

A hallmark of endothelial function is the synthesis and release of nitric oxide (NO), which provides local regulation of vasomotor tone and anti-thrombotic actions [19]. eNOS is constitutively expressed in vascular endothelium. The polymorphism in intron 4 and G894T polymorphisms of eNOS have been demonstrated to reduce NO levels in human plasma [20]. Thus, polymorphisms of eNOS gene and resultant reduction of NO synthesis should lead to vascular abnormalities. Indeed, many studies have observed associations between genetic polymorphisms in the eNOS gene and vascular diseases, including coronary artery disease or myocardial infarction, hypertension, stroke, and renal diseases [21]. ANFH is a phenomenon involving the disruption of vascular supply to the femoral head [22]. However, the role of eNOS in the vascular pathogenesis of ANFH is currently under investigation. Current evidence suggests that intravascular coagulation and microcirculatory thrombotic occlusion may be responsible for the decrease in vascular supply to the femoral head in non-traumatic osteonecrosis. For example, arteriolar and other intravascular thromboses have been found in large numbers of osteonecrotic femoral heads [23]. Elevated levels of fibrinopeptides and fibrin degradation products have been observed in patients with osteonecrosis [23], [24]. It is therefore not surprising that eNOS polymorphism is associated with the pathogenesis of ANFH.

We acknowledge that there are several limitations to this study: 1) The eNOS activity was not measured. Although previous studies have demonstrated that the 27-bp repeat polymorphism in intron 4 is associated with the altered function of eNOS [11], the basal NO metabolite levels in plasma were lower in subjects with eNOS 4a/a genotype than in those with eNOS 4b/a and eNOS 4b/b genotype. However, in this study, the frequency of eNOS b/a, but not a/a and b/b genotype was significantly higher in the idiopathic subgroup of ANFH patients compared to controls. Further studies are required to determine whether the association of 4a/b genotype with ANFH is due to the decrease in eNOS activity. Currently, there are no reports available on the association between eNOS polymorphism and measured eNOS activity in the plasma or hip joint of ANFH patients. 2) The combined effects of polymorphism in intron 4 and G894T polymorphisms in the pathogenesis of ANFH were not analyzed due to the relatively small sample size. 3) The frequency of 4a allele and a/a genotype was extremely low although this was also true in other studies. Thus, the bias due to small sample size cannot be fully excluded.

In conclusion, microsatellite polymorphism in intron 4 and G894T polymorphisms in exon 7 of eNOS are associated with idiopathic ANFH in Chinese patients, which support the role of eNOS in the pathogenesis of idiopathic ANFH. To firmly establish the relationship between the eNOS polymorphisms and idiopathic ANFH, further large-scale, multicenter studies of patients are needed in Han Chinese and other populations.
